# Driving-Related Cognitive Abilities Prediction Based on Transformer’s Multimodal Fusion Framework

**DOI:** 10.3390/s25010174

**Published:** 2024-12-31

**Authors:** Yifan Li, Bo Liu, Wenli Zhang

**Affiliations:** Faculty of Information Science and Technology, Beijing University of Technology, Beijing 100124, China; yifanli@emails.bjut.edu.cn (Y.L.); liu_bo@emails.bjut.edu.cn (B.L.)

**Keywords:** biosignals, driving-related cognitive abilities, multimodal, driving safety

## Abstract

With the increasing complexity of urban roads and rising traffic flow, traffic safety has become a critical societal concern. Current research primarily addresses drivers’ attention, reaction speed, and perceptual abilities, but comprehensive assessments of cognitive abilities in complex traffic environments are lacking. This study, grounded in cognitive science and neuropsychology, identifies and quantitatively evaluates ten cognitive components related to driving decision-making, execution, and psychological states by analyzing video footage of drivers’ actions. Physiological data (e.g., Electrocardiogram (ECG), Electrodermal Activity (EDA)) and non-physiological data (e.g., Eye Tracking (ET)) are collected from simulated driving scenarios. A dual-branch Transformer network model is developed to extract temporal features from multimodal data, integrating these features through a weight adjustment strategy to predict driving-related cognitive abilities. Experiments on a multimodal driving dataset from the Computational Physiology Laboratory at the University of Houston, USA, yield an Accuracy (ACC) of 0.9908 and an F1-score of 0.9832, confirming the model’s effectiveness. This method effectively combines scale measurements and driving behavior under secondary tasks to assess cognitive abilities, providing a novel approach for driving risk assessment and traffic safety strategy development.

## 1. Introduction

In modern society, driving has become an integral part of daily life. According to a survey, 94% of traffic accidents are caused by driver-related factors [1]. Beyond mastering vehicle operation skills, drivers must also possess a wide range of complex cognitive abilities to handle various traffic situations and emergencies [2,3,4,5]. Therefore, gaining a comprehensive understanding of the cognitive abilities involved in driving has significant theoretical and practical value. This knowledge is critical for assessing whether drivers are capable of safely operating vehicles, helping them understand their driving performance, and enabling governments to conduct more scientific medical examinations.

In recent years, cognitive science and neuropsychology have been introduced into driving research [6,7,8]. By studying cognitive processes such as perception, response, and memory in driving, researchers like Moran et al. [9] have proposed a theoretical framework that divides driving behavior into several interrelated cognitive sub-capacities, including attention allocation, rapid response, processing speed, working memory (WM) capacity, reaction ability, judgment, anticipatory ability, and perceptual skills. A deficiency in any of these abilities can lead to serious traffic accidents and heightened driving risks. Young drivers (aged 17–25) and older drivers (aged 60 and above) are particularly considered high-risk groups [10]. Younger drivers, especially those newly licensed, may encounter unfamiliar driving challenges and exhibit impulsivity, leading to relatively weaker cognitive driving abilities [11,12]. In older adults, cognitive abilities tend to decline with age [13,14].

Most current studies on driving-related cognitive abilities assessments focus on specific cognitive components such as attention [15,16,17,18], reaction speed [19,20,21], working memory (WM) capacity [22,23,24], or perceptual ability [9,25,26]. For example, Zatmeh-Kanj and Toledo [16] proposed the Gipps’ Model (GM) for car-following based on vehicle data, a classical model used to assess driving attention. In this study, participants were tasked with following a lead vehicle that randomly changed speeds while maintaining a consistent distance. Data including average speed, speed variability, acceleration, deceleration times, and distance to the lead vehicle were used as inputs for the GM model to evaluate driver attention levels. Similarly, Chen et al. [17] analyzed behaviors that distract attention while driving, examining four secondary tasks: answering math questions, solving problems, texting, and normal driving. By observing changes in drivers’ behavior and physiological data during these tasks, they developed a neural architecture search network to perform detailed analyses of attention-diverting activities. Mohammed et al. [18] proposed a semi-supervised lightweight vision transformer method based on pseudo-labeling, which effectively detects driver distraction behaviors in natural driving environments by utilizing both unlabeled and a small amount of labeled data for model training. This approach achieved an average accuracy of 95.43% on the StateFarm dataset. Stanisław et al. [20] examined reaction time by measuring dynamic vehicle parameters such as response time for the accelerator, brake, and steering inputs, presenting the data as a Time-To-Collision (TTC) function. Their study demonstrated that these parameters effectively evaluated driver reaction ability under various conditions, providing a scientific basis for enhancing driving safety. Additionally, Hajinoroozi et al. [21] integrated Electroencephalogram (EEG) data with reaction times to assess drivers’ states. Their findings suggested that a reaction time (RT) of ≤0.7 s indicates good driving performance, whereas an RT of ≥2.1 s reflects poor performance. EEG signals helped distinguish between drivers with strong and weak driving capabilities. Broadbent et al. [24] found that by setting tasks with varying levels of working memory (WM) demands and employing biometric methods such as Functional Near-Infrared Spectroscopy (FNIRS) and eye-tracking, it is possible to effectively evaluate drivers’ WM capacity and its impact on driving performance. Their findings indicate that WM capacity significantly influences drivers’ reaction times and overall performance, particularly under high-load tasks. In a study analyzing the effect of perceptual abilities on driving, Moran et al. [9] employed newly developed testing methods that included the accuracy of recognizing potential hazards, the reaction time required to anticipate these hazards, and the time needed to take action to avoid them. They recommended incorporating cognitive function assessments into graduated licensing systems to help identify young drivers whose cognitive skills may be insufficient for safe driving. Moghaddam and Ayati [27] found that Type A drivers, characterized by competitiveness and a sense of urgency, are more prone to high-risk driving behaviors and have higher accident rates. In contrast, Type B drivers, who tend to exhibit more relaxed and patient driving styles, are generally safer. Therefore, when developing traffic safety strategies and interventions, personality traits should be considered.

These studies primarily analyze driver distraction, reaction capabilities, and other factors by combining secondary tasks with subjective and objective data measurement methods. However, they focus on individual components of cognitive driving abilities without fully considering the complexity of the driving process and the multifaceted nature of cognitive abilities.

Some subsequent studies have attempted to analyze the effects of driving-related cognitive abilities holistically and comprehensively, for example, Depestele et al. [28] set up various driving scenarios to assess cognitive abilities and conduct driving simulator tests. They found that cognitive capabilities are significantly positively correlated with driving performance, both in elderly and young individuals. Ledger et al. [29] targeted two groups with a higher risk of collisions, namely young and elderly drivers, using various cognitive measurement tools such as the Rey Complex Figure Test (CFT), Trail Making Test (TMT), and Mini-Mental State Examination (MMSE). Through statistical analysis, they observed the relationship between drivers’ cognitive abilities and driving performance. They found that, regardless of age, drivers’ cognitive abilities are a crucial factor in assessing driving performance. Mullen et al. [30] recorded the performance of elderly drivers in driving behaviors such as turning, braking, and accelerating using a driving simulator and statistical analysis methods. Additionally, they employed a series of cognitive measurement scales to assess the cognitive abilities of elderly drivers, including short-term memory, spatial working capacity, processing speed, and attention. The study results indicate that driving-related cognitive abilities are an important basis for assessing the driving performance of elderly drivers. However, Stolwyk et al. [31] found that the correlation between on-road driving performance and standard neuropsychological tests was not significant, especially for healthy individuals without significant cognitive impairment. The studies mentioned above demonstrate that evaluating driving-related cognitive abilities through various scales, task scenarios, and driving simulators to observe driver performance in behaviors such as steering, braking, and acceleration is effective. Regardless of age, there is a significant positive correlation between cognitive abilities and driving performance. However, these studies mainly use statistical methods to analyze data relationships, which perform inadequately when handling high-dimensional data and fail to fully extract common features.

Based on the research surveyed above, it is found that current studies mostly investigate the correlation between a single element of driving-related cognitive abilities and driving performance. Even when some studies attempt to integrate these driving-related cognitive abilities, they have only used scales or have not comprehensively synthesized all aspects of driving-related cognitive abilities. In addition, most of the methods analyzed in these studies use machine learning or statistical models, although machine learning has advantages in speed and efficiency, its performance is heavily dependent on the researcher’s expertise and the selection of data features. In the processing of high-dimensional and complex data, feature selection and data preprocessing require extensive specialized knowledge. Therefore, it is necessary to further utilize advanced modeling algorithms to comprehensively explore the integrated impact of different driving-related cognitive abilities on driving behavior.

In recent years, with the continuous emergence of big data and the improvement of computational capabilities, sensor technology has also been evolving rapidly. Sensors used for driving behavior analysis include physiological and non-physiological measurement sensors. Physiological or neurophysiological measurements include ECG, EEG, EDA, galvanic skin response (GSR), and respiratory rate [32]. Non-physiological measurement sensors include vehicle signals such as speed, acceleration, lane deviation, and braking force, eye trackers measuring eye displacement in the X/Y directions, and depth cameras for capturing facial emotional signals [33,34,35,36]. The continuous advancement of these sensor technologies has led to a more comprehensive acquisition of driving behavior data, which provides a richer source of information for assessing a driver’s cognitive ability to drive. At the same time, this poses a challenge to the processing of such multi-source data. The large amount of multi-source data makes it challenging to obtain high-dimensional features. Therefore, the distributed and hierarchical feature characteristics of deep learning have emerged as effective methods for processing these data and have found wide application in driving behavior assessment. For this purpose, Mou et al. [32] proposed a novel dual-channel feature extraction model CNN (Convolutional Neural Network) -Trans-SA based on CNN and Transformer. This model takes the eye, vehicle, and physiological data as input features to perform fine-grained classification of driver distraction levels, including normal driving, cognitive distraction, emotional distraction, and sensory-motor distraction. The classification ACC of the model reached 99.802%. Arvin et al. [33] employed the concept of volatility by integrating a Data Acquisition System (DAS) with various sensors such as front and in-car cameras, front sensors, and accelerometers to collect vehicle motion, cabin video, and surrounding environment data streams. They utilized a combination of a one-dimensional 1D-Convolutional Neural Network (1D-CNN) and Long Short-Term Memory (LSTM) to build a 1DCNN-LSTM model for processing these data. This model extracts local dependencies and volatility from time-series data to achieve distraction classification of drivers, achieving the model classification ACC of 95.45% in their study. Mou et al. [34] proposed an attention-based framework for multimodal fusion of driver stress detection. The framework utilizes an attention-based CNN and LSTM model to fuse non-invasive data, including eye data, vehicle data, and environmental data. With this model, features can be automatically extracted from each modality and features from different modalities can be weighted through a self-attention mechanism to increase attention to important features. The method achieves the model average classification ACC of 95.5%. Arefnezhad et al. [35] proposed a combined CNN and Recurrent Neural Network (RNN) based method for driver sleepiness detection. The method takes five vehicle-based measurements as inputs to the network, including lateral deviation from the road centerline, lateral acceleration, traverse angular velocity, steering wheel angle, and steering wheel speed. In the RNN layer, LSTM and Gated Recurrent Unit (GRU) are used. By combining with CNN (CNN-LSTM), the method achieved the highest model classification ACC of 96.0%. Vyas et al. [36] proposed a transformer-based end-to-end driver behavior classification framework called Trans-DBC that aims to classify unsafe driving behaviors. The framework enables the analysis of driver behavior by effectively using accelerometer, gyroscope, and GPS multivariate time-series data combined with learning short-term and long-term time dependencies. The results of the study show that the framework can achieve 95.38% classification ACC.

The above research indicates that multimodal data provides rich feature information. To effectively predict driving-related cognitive abilities, it is essential to maximally extract the common features from these data. This requires designing an appropriate network model to reduce the interference caused by different distribution characteristics at the data source level, mapping the features into a high-dimensional space, and maximally integrating the common features across all data to effectively predict driving-related cognitive abilities. Table 1. presents a summary table form of the current state of research related to the assessment of driving behavior mentioned above, which includes the research subjects, the signals used, the assessment methods, and the experimental results.

In summary, although existing research has explored various cognitive factors related to driving performance in different driving scenarios, most studies are limited to the analysis of a single cognitive element, which leads to an incomplete understanding of the factors influencing driving behavior. This study systematically analyzes the components of driving-related cognitive abilities and their interactions with driving behavior from three dimensions: cognitive decision-making, vehicle control, and psychological state. By improving the model, we are able to extract key common features from multimodal sensor data, allowing for a more accurate and comprehensive evaluation of driving-related cognitive abilities. Therefore, this study proposes a method for assessing driving-related cognitive abilities based on multimodal data. This method initially introduces a comprehensive way to quantify driving-related cognitive abilities. By utilizing driving behavior data across different scenarios, it extracts ten fundamental cognitive elements comprising driving-related cognitive abilities: attention allocation, processing speed, working memory capacity, reaction ability, judgment, anticipation, perception, stress resistance, anxiety level, and Type A/B personality traits, further refining the characterization of driving-related cognitive abilities. Concurrently, a predictive method for driving-related cognitive abilities based on multimodal driving data is proposed. This approach aims to identify relationships between sensor data and driver cognitive abilities, utilizing the Transformer self-attention mechanism and multimodal fusion techniques to predict drivers’ cognitive capabilities. The proposed method in this paper contains the following main contributions:

- Introduction of a Multidimensional Cognitive Ability Evaluation System: This study defines and quantifies cognitive factors affecting driving safety from three dimensions—cognitive decision-making, vehicle control, and psychological state. It extracts and quantifies multiple cognitive ability indicators related to driving, thereby providing a comprehensive assessment of drivers’ cognitive capabilities and addressing the limitations of previous studies that focused solely on single cognitive dimensions.
- Development of a Multimodal Data Feature Extraction Model: To address the challenge of effectively extracting common features between multimodal data in traditional models, this study proposes a dual-branch Transformer network. The dual-branch structure eliminates interference between input data and effectively learns high-dimensional features of the data. Additionally, for different data sources, a dynamic weight allocation-based feature fusion mechanism is introduced, enhancing the feature representation ability and predictive performance of the final model.

## 2. Materials and Methods

### 2.1. The Datasets

This study utilizes the multimodal driving dataset published by Salah Taamneh et al. [37], which comprises 68 participants, including 34 young participants (aged 18–27 years, mean = 22.41 ± 1.7 years) and 34 elderly participants (aged 61–86 years, mean = 69.97 ± 6.6 years). The dataset was collected using a driving simulator manufactured by Realtime Technologies, Inc. (located in Royal Oak, MI, USA). During the simulated driving task, participants were required to drive along a 10.9 km straight section of a four-lane highway with a speed limit of 70 km/h. There were two dedicated lanes in each direction, with participants driving in the right lane (R), while the opposite lane had heavy traffic (more than 12 vehicles per kilometer). The left lane (L) was under construction, and traffic signposts were located on both sides of the right lane (R). During this task, participants had to complete various driving tasks, including secondary and failure-driving tasks, which involved distracted driving scenarios such as answering questions, texting, etc., while also facing challenges like construction zone crossings and speed limit signs. In the failure-driving task phase, a vehicle malfunction caused an unintended acceleration event before the traffic light at an intersection turned green, propelling the car forward and causing a collision with another vehicle entering the intersection. Throughout these simulated driving tasks, a range of sensors recorded both physiological and non-physiological data of the drivers for analysis.

1. Physiological sensors include the following:

- Skin galvanic response signals were collected using the Shimmer3 GSR sensor (Shimmer, Dublin, Ireland) with a sampling frequency of 25 Hz to record the driver’s physiological arousal state.
- Heart rate and respiratory rate were collected using the Zephyr BioHarness 3.0 (Zephyr Technology, Annapolis, Maryland) with a sampling frequency of 1 Hz.
- The Tau 640 long-wave infrared (LWIR) camera (FLIR Commercial Systems, Goleta, California) with a frame rate of 7.5 fps was used to capture perspiration signals around the nose area.

2. Non-physiological sensors include the following:

- Eye movement parameters, such as gaze position and pupil diameter, were recorded using the faceLAB system (Seeing Machines, Canberra, Australia) with a sampling frequency of 25 Hz to analyze the driver’s attention state.
- Data on vehicle speed, acceleration, braking, steering, lane deviation, and lane position were collected using the driving simulator manufactured by Realtime Technologies, Inc. (Royal Oak, Michigan) with a sampling frequency of 58.8 Hz to analyze vehicle control state.
- Emotional state data were recorded using the HD Pro Webcam C920 (Logitech, Newark, CA, USA) monochrome zoom camera with a frame rate of 15 fps. The data were pre-processed and analyzed to assess changes in the driver’s emotional state.

### 2.2. General Structure of the Framework

**Figure 1:**
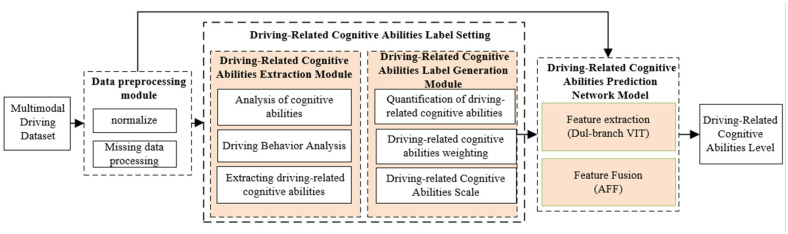
Overall technical framework.

In this study, a Transformer-based Dual-branch multimodal feature fusion network model is proposed for realizing the prediction of a driver’s driving-related cognitive abilities. Figure 1. shows the general technical framework of this paper.

In Figure 1, the shaded blocks are the main innovations of this study. In this study, the components of driving-related cognitive ability are extracted and quantified by the Driving-Related Cognitive Abilities Extraction Module, and the driving-related cognitive ability labels are generated by the Driving-Related Cognitive Abilities Label Generation Module by assigning weights to each component to generate weighted scores. Meanwhile, this study proposes a Dual-branch transformer network in the Driving-Related Cognitive Abilities Prediction Network Model, based on the VIT network, the input features are sent to different branches for feature extraction, and the extracted features are fused with the AFF network, which enhances the feature extraction capability of the model. The overall technical framework of this paper consists of the following four main modules.

- Data Preprocessing Module: Due to the complexity and diversity of the data contained in multimodal driving data, the raw data need to be preprocessed before feature extraction. This module initially removes unstable data recorded before the start of the experiment and addresses any missing data. It also eliminates motion artifacts caused by actions such as blinking and selects an appropriate sliding window to provide sufficient samples for training.
- Driving-Related Cognitive Abilities Extraction Module: This module is designed to assess the performance of drivers during various secondary tasks and malfunction handling tasks. It extracts and quantitatively represents the components of driving cognitive-related abilities based on this performance.
- Driving-Related Cognitive Abilities Label Generation Module: This module assigns weights to the quantified components of driving-related cognitive abilities based on their importance and performs a weighted sum. Labels for driving-related cognitive abilities are then generated based on the weighted scores.
- Driving-Related Cognitive Abilities Prediction Network Model: After the above modules, clean driving data and labels for driving-related cognitive abilities were obtained. At this point, an appropriate network model is required to extract and fuse features from the processed data, enabling precise prediction of driving-related cognitive abilities.

In this paper, the above four modules are described in detail in Section 2.3, Section 2.4 and Section 2.5.

### 2.3. Data Preprocessing Module

Before performing the prediction task, the 15-dimensional multimodal driving data needs to be preprocessed to eliminate noise, missing values, and outliers from the data to ensure ACC and completeness. Consider the input X is an n-dimensional time-series signal, and using Min–Max normalization, X is normalized between 0 and 1 to obtain $X^{\prime }$. The normalization formula is as follows:

(1) $$X^{\prime }=\frac{X-X_{min}}{X_{max}-X_{min}}$$

In this study, a sliding window approach with overlap was used to partition the normalized multimodal driving data into appropriate sizes. These sliding window sizes and step sizes were chosen based on the results of previous studies on multimodal driving behavior analysis [17,21,32,33]. Time series of fixed length and width were generated through the sliding window approach. These sequences were designed to overlap between neighboring windows to maintain continuity in the window sequences and provide enough samples for training, enabling the network to better acquire features. The sequences generated through the windowing method are upscaled into uniformly sized 2D image data, which are aligned with the original labels and used as data for model training.

To address the issue of missing data in multimodal driving datasets, particularly the asynchrony observed among multiple sensors during the initial recording phase (i.e., the first 1–10 s), this study initially excluded these asynchronous data records. Subsequently, linear interpolation was employed to impute the missing values for the synchronized data, thereby ensuring the dataset’s high quality and completeness. This ensures that the data can be effectively utilized for network training.

### 2.4. Driving-Related Cognitive Abilities Label Setting

Driving is a process that involves the brain’s rapid recognition of external information, decision-making, and vehicle control through actions such as steering or braking. This process is also influenced by the driver’s psychological state. To deeply investigate and quantify the key factors affecting the driving process, this study designed a set of driving-related cognitive abilities assessment methods, aiming to quantitatively assess the performance of drivers in various aspects of thinking decision-making, vehicle maneuvering, and changes in psychological state during driving activities.

Driving-related cognitive abilities refer to the driver’s ability to process information and make decisions, precisely control the vehicle, and effectively regulate psychological states during the driving process. Driving-related cognitive abilities can reflect the driver’s reception and processing of information, rapid execution of decision-making, and changes in psychological state. This study comprehensively examined the changes in driving behavior and classified driving-related cognitive abilities into three dimensions based on the different needs of driving tasks: thinking decision-making, vehicle manipulation, and psychological regulation, which contain ten basic elements that make up driving-related cognitive abilities. The specific details are as follows:

- Cognitive decision-making dimension: This explores how drivers process received traffic information and quickly make decisions based on this information. It includes the assessment of drivers’ attention allocation, working memory, and anticipation abilities.
- Vehicle control dimension: This directly reflects how drivers translate decisions into vehicle operations, covering assessments of drivers’ processing speed, reaction capability, judgment, and perceptual skills.
- Psychological regulation dimension: This examines how drivers influence decision-making and action execution through the regulation of their psychological state, assessing drivers’ stress resilience, anxiety levels, and Type A/B personality traits.

To quantitatively assess these components of driving-related cognitive abilities, this study integrates subjective measures (e.g., questionnaires) with objective measures (e.g., behavioral analysis). Subjective measures reflect the self-assessed abilities of the driver, while objective measures provide data on the actual driving performance of the driver. This study describes in detail how to extract and quantify the components of driving-related cognitive abilities based on the video data in Section 2.4.1. In Section 2.4.2, a systematic assessment method is designed to assign weights to the quantified indicators and weight the scores, and the driving-related cognitive ability is finally graded as “Insufficient”, “Fair”, “Good “ and “Excellent”.

#### 2.4.1. Driving-Related Cognitive Abilities Extraction Module

This study analyzes the performance of drivers in various tasks recorded in driving videos, observing their performance in tasks that require focused attention, rapid response, accurate decision-making, and handling complex situations to extract the components of driving-related cognitive abilities. In driving cognitive ability assessment, elements such as attention allocation and perception ability do not exist in isolation, and there may be differences in cognitive abilities involved in normal driving and dangerous situations [38]. Additionally, these driving-related cognitive abilities have varying priorities in different task scenarios, emphasizing different aspects. The key cognitive abilities involved in driving can be extracted through different task scenarios. For example, multiple studies [6,39,40] have pointed out that while both reaction ability and processing speed involve reaction time, reaction ability mainly refers to the driver’s braking time in emergencies, while processing speed refers to the driver’s information processing efficiency when faced with complex road conditions. Perception ability involves the driver’s awareness of traffic signs, vehicles, and the relative position of the surrounding environment. These reflect different cognitive processes in emergencies and when handling secondary tasks. The specific methods for extracting the ten elements of driving-related cognitive abilities within the three dimensions are presented in Table 2.

Table 2 lists the foundational data sources for each driving-related cognitive ability. The dimension features corresponding to each data source are extracted through specific driving task scenarios. For example, reaction ability is evaluated through a simulated emergency braking task, while processing speed is assessed by the completion time of secondary tasks in complex road conditions. By analyzing task performance across different scenarios, we effectively extract the driver’s performance across various cognitive ability dimensions. Due to the difference in the experimental setup, members of group A in the faulty driving task needed to complete additional tasks, thus creating a bias in the calculation of reaction time. To eliminate this effect, the study used the method of time compensation [20]. It standardized the reaction time data for the two subject groups. This was achieved by using the difference between the average reaction times of groups A and B as compensation. The method of reaction time compensation is calculated by Equation (2).

(2) $$CRT=\frac{\left({RT}_{A}-{RT}_{B}\right)}{N}$$

where CRT denotes compensated reaction time, ${RT}_{A}$ denotes the group a reaction time, ${RT}_{B}$ denotes the group’s reaction time, and N denotes the total number of people.

To accurately quantify driving-related cognitive abilities, this study follows these steps:

- First, directly quantifiable indicators such as reaction times and scale scores are kept in their original values for standardization.
- Second, qualitative behavioral performances, such as the degree of collision, are numerically processed. For example, ’no collision’ is marked as 0, and ’collision’ is marked as 1. This step completes the numerical transformation of all data.
- Finally, the formula $Z=\frac{\left(X-\mu \right)}{\sigma }$ is applied to standardize the numerically transformed data.

where Z denotes normalization, X denotes indicator value, μ denotes sample mean, and σ denotes sample standard deviation.

These steps produce quantified driving-related cognitive abilities, which are then used for generating labels in Section 2.4.2.

#### 2.4.2. Driving-Related Cognitive Abilities Label Generation Module

To map driving-related cognitive abilities to different levels, the data were first processed using the G-Rules-IQR (Gaussian-Rules Interquartile Range, abbreviated as G-Rules-IQR) method [41] to adjust the distribution closer to a normal distribution. Then, based on the first quartile (Q1), the second quartile (Q2, median), and the third quartile (Q3) of the data, all scores were mapped into four ranges. These four ranges represent different levels of driving abilities: Excellent (above Q3), Good (between Q2 and Q3), Fair (between Q1 and Q2), and Insufficient (below Q1). Converting the quantified driving-related cognitive abilities into a driving-related cognitive abilities label may be accomplished by steps 1–3.

- Analysis of the Importance of Components of Driving-Related Cognitive Abilities: Anstey et al. [42] found that thought decision-making directly reflects the driver’s response to environmental changes and decision-making efficiency, hence it is of the highest importance. Vehicle control reflects the driver’s actual execution ability in controlling the vehicle and responding to road conditions, making it the second most important. Psychological adjustment, while having a non-negligible impact on driving safety, has the least influence compared to thought decision-making and vehicle control abilities that directly determine driving response and vehicle control, thus it is of the lowest importance.
- Generation of Weighted Scores for Components of Driving-Related Cognitive Abilities: Based on step 1, the importance of three dimensions of driving-related cognitive abilities from high to low are: Cognitive decision-making (dim.1), vehicle control (dim.2), and psychological regulation (dim.3). The weights for each cognitive ability dimension were determined using the Principal Component Analysis (PCA) method [43]. Specifically, PCA was applied to the indicators extracted in Table 2 to identify their linear combination coefficients in each principal component. Based on the variance contribution rate of each principal component, the weights $W_{1}$, $W_{2}$, $W_{3}$ were calculated and assigned to dim.1, dim.2, and dim.3, respectively. The driving-related cognitive abilities score (Score for short) is obtained through weighting. $W_{i}$ and Score can be calculated using Equations (3) and (4). The distribution of driving-related cognitive abilities weights is shown in Table 3.

(3) $$W_{i}=\sum_{k=1}^{K}\;L_{ik}\cdot V_{k}$$

(4) $$Score=\sum_{i=1}^{3}\;W_{i}\cdot dim.i$$

where $L_{ik}$ denotes the loading of the ith variable on the kth principal component, $V_{k}$ denotes the ratio of variance explained by the kth principal component, and K denotes the number of principal components selected.

**Table 3:** Driving-related cognitive abilities weight distribution table.

Driving-Related Cognitive Abilities Dimensions	$\mathrm{Importance}\;\mathrm{Weight}\;W_{i}$	Weighting
Thinking Decision Making	$W_{1}$	0.3898
Vehicle Handling	$W_{2}$	0.3182
Mental State	$W_{3}$	0.2920

- Generation of Driving Cognitive-Related Ability Labels: Driving-related cognitive abilities labels are divided into four levels, 0–3, representing four levels of driving-related cognitive abilities: Insufficient, Fair, Good, and Excellent. These four levels are determined based on the Score derived from Equation (4). The calculation of the Score incorporates the weighted values of ten components of driving-related cognitive abilities, reflecting the comprehensive level of driving-related cognitive abilities. Since these components of driving-related cognitive abilities have different distribution characteristics in terms of centrality and dispersion, the weighted Score results in a multivariate composite distribution. To effectively categorize Scores into levels, this study employs the G-Rules-IQR method. This method does not rely on the normal distribution properties of the data and can determine the score thresholds $Q_{i}$ for “Score” by calculating the interquartile range (IQR). This method maps the continuous “Score” to four discrete intervals {[${Score}_{min}$, $Q_{1}$), [$Q_{1}$, $Q_{2}$), [$Q_{2}$, $Q_{3}$), [$Q_{3}$, ${Score}_{max}$]}, achieving the classification of driving-related cognitive abilities levels and thus generating driving-related cognitive abilities labels. The $Q_{i}$ can be calculated using Equation (5).

(5) $$Q_{i}=\mu +\left(\sigma \times z_{p_{i}}\right)\;(i=1,2,3)$$

where $Q_{i}$ corresponds to the index of the quartile ($Q_{1}$ denotes the first quartile, $Q_{2}$ denotes the second quartile, $Q_{3}$ denotes the third quartile), μ denotes the mean of the sample Score, σ denotes the standard deviation of the sample Score, and $z_{p_{i}}$ denotes the different percentiles of Score.

The mapping relationship between the driving-related cognitive abilities labels and Score is calculated by Equation (6).

(6) $$M_{score}=\left\{\begin{array}{l} 0,\;\;\;\;\;\;\;\;\;{\mathrm{Score}}_{min}\leq Score<Q1\\ 1,\;\;\;\;\;\;\;\;\;Q1\leq Score<Q2\\ 2,\;\;\;\;\;\;\;\;\;Q2\leq Score<Q3\\ 3,\;\;\;\;\;\;\;\;\;Q3\leq Score\leq {\mathrm{Score}}_{max} \end{array}\right.$$

where 0–3 denotes the driving-related cognitive abilities labels corresponding to the four levels of driving-related cognitive abilities: Insufficient, Fair, Good, and Excellent, respectively. ${\mathrm{Score}}_{min}$ and ${\mathrm{Score}}_{max}$ denotes the minimum and maximum values, respectively, and Q1–Q3 denotes the Score thresholds.

Driving-related cognitive abilities labels were obtained through the processing of the above two modules. The subjects were regrouped according to the labels and the labeled data were fed into the Section 2.5 Driving-related cognitive abilities Prediction Network Module for final driving-related cognitive abilities prediction.

### 2.5. Driving-Related Cognitive Abilities Prediction Network Model

In the field of driving behavior analysis, effectively extracting and fusing multimodal data from different sensors presents a challenge. The common practice in current research is to directly concatenate the time-series data of different modalities, such as respiratory rate, eye movement coordinates, and vehicle dynamics, without considering the inherent differences in feature expression and diversity among the input data. This approach can easily lead to confusion of information and loss of key features. Furthermore, when dealing with complex driving behavior data, traditional data processing and feature extraction methods based on rules or manually designed features struggle to effectively capture the high-dimensional nonlinear characteristics of the data, thereby affecting the ACC of predictions.

**Figure 2:**
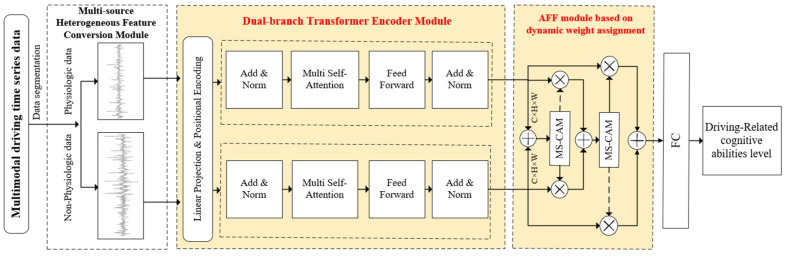
Multimodal feature fusion network model.

To address the above problems, this study proposes an improved Dual-branch Transformer network structure. It aims to effectively extract and fuse temporal features from heterogeneous data from multiple sources, such as physiological and non-physiological, to adapt to the needs of driving-related cognitive abilities prediction. The structure addresses the differences in the feature expression and feature diversity of multi-source heterogeneous data by employing two parallel encoders, each dedicated to the extraction of physiological and non-physiological features, respectively. This approach resolves the issue of consistency in the feature expression of input data. Moreover, to further weigh the relative importance of the features extracted by physiological and non-physiological encoders, the Attentional Feature Fusion (AFF) algorithm [44] is introduced in the feature fusion stage of this study. This algorithm optimizes the fusion of physiological and non-physiological features through a dynamic weight allocation mechanism and precisely adjusts the relative contribution of these two features in the information representation process, thereby effectively enhancing the ACC of the model in predicting driving-related cognitive abilities. The network model structure proposed in this study is illustrated in Figure 2 and is based on the ViT [45] design. It comprises three main components: a Multi-source Heterogeneous Feature Conversion Module, a Dual-branch Transformer Encoder Module, and an AFF module based on dynamic weight assignment.

- Multi-source Heterogeneous Feature Conversion Module: This module is responsible for separating physiological data from non-physiological data from multi-modal driving time-series data, and converting them from the original 2D time-series data to 2D image data, which meets the needs of the dual-branch Transformer encoder module for feature extraction from the input data.
- Dual-branch Transformer Encoder Module: This module adopts an improved dual-branch structure, which allows physiological and non-physiological data to be feature extracted by independent transformer encoders. This dual-branch structure allows the physiological branch to focus on capturing changes in the detailed features of the physiological signals in the short term, and the non-physiological branch to focus on capturing changes in the long-term features such as vehicle operating modes. A self-attention mechanism is used within each branch to optimize the capture of dynamic time-series data features and enhance the feature extraction capability of the model for multimodal driving time-series data.
- AFF module based on dynamic weight assignment: after physiological and non-physiological features are extracted by the Dual-branch Transformer encoder module, the attention weights of global and local feature channels are extracted by the AFF module using global pooling and point-by-point convolution, where global pooling is in charge of capturing the global feature information and point-by-point convolution is used for extracting and strengthening local feature details. Finally, the performance of the prediction model is further optimized by feature fusion.

#### 2.5.1. Multi-Source Heterogeneous Feature Conversion Module

This module processes the input multimodal driving time-series data, which are categorized into physiological and non-physiological data according to their physiological properties. The input data are in the form of $X\in R^{H\times W}$, where H and W represent the height and width of the data, respectively. By splitting and reshaping operations, X is split into physiological data $X_{p}\in R^{H\times W\times C}$ and non-physiological data $X_{np}\in R^{H\times W\times C}$, where C is the number of channels. The processed data are converted into 2D image format for feature extraction by the Dual-branch Transformer encoder module.

In order to adequately represent the temporal characteristics of the time-series data, the sliding window method is used to generate the set of temporal windows D. Each temporal window $X_{i}\in R^{H\times W}(1\leq i\leq m)$ denotes the ith window. For physiological data $X_{p}$ there are timing windows $D_{p}$ denoted as:

(7) $$D_{p}=\left\{\left(X_{1}^{P},y_{1}^{p}\right),\left(X_{2}^{P},y_{2}^{p}\right),\ldots ,\left(X_{i}^{P},y_{i}^{p}\right)\right\},1<i<m$$

where $X_{i}^{P}$ denotes the ith window, and $y_{i}^{p}$ is the corresponding category label for the temporal window. The set of temporal windows $D_{p}$ contains m windows and all temporal windows will be used as model inputs for further feature extraction.

#### 2.5.2. Dual-Branch Transformer Encoder Module

For nonlinear time-series feature extraction of multimodal driving time-series data, this study proposes a Dual-branch Transformer encoder module, which adopts two parallel Transformer encoder structures for feature extraction of physiological data $X_{p}$ and non-physiological data $X_{np}$ processed by a multi-source heterogeneous feature transformation module, respectively, so that the module not only focuses effectively on the changes of the detailed features of the physiological parameters, such as heart rate, EDA, and other physiological parameters but also captures the changes of the long-term dynamic features of the non-physiological data regarding the operating modes of the vehicle.

First, the physiological data $D_{p}$ undergoes a linear projection layer, which maps the raw data containing four dimensions, including skin EDA, heart rate, respiratory rate, and nasal sweating signals, into a high-dimensional feature space, effectively capturing the complex high-dimensional features of the physiological data. Then, to preserve the temporal and label information of each window, we apply position encoding by adding sinusoidal and cosine functions with a fixed frequency. The position encoding introduces an additional positional marker vector $E_{pos}$ for each temporal window, helping the model recognize the positional information of the sequential data. The label information is incorporated by adding a classification marker vector $Z_{cls}$ to each window. Finally, the temporal branch feature vector set $X_{p}^{0}$ is generated. The specific implementation of the linear projection and position encoding can be referred to in Equation (8).

(8) $$X_{P}^{0}=[Z_{cls}^{p};d_{1}^{p}E^{p};d_{2}^{p}E^{p};\ldots ;d_{m}^{p}E^{p}]+E_{pos}^{p}$$

where $X_{p}^{0}$ denotes the initial features after linear projection and positional coding process, where $E^{p}$ is the linear projection matrix, $d_{1}^{p}$, $d_{2}^{p}$ …, $d_{m}^{p}$ are the temporal slice vectors.

After processing, the initial features $X_{P}^{0}$ are fed into a Transformer encoder composed of a multi-head self-attention (*MSA*) mechanism and a feed-forward network (*FFN*). Each feed-forward network consists of two fully connected layers, with a dropout layer applied after each layer. The dropout rate is set to 0.2.

The encoded features $X_{p}^{0}$ were obtained and next $X_{p}^{0}$ is fed into a Transformer encoder consisting of a Multi-head Self-Attention (MSA) Mechanism and a Feedforward network (FFN). The *MSA* allows the model to encode a feature at one position taking into account the features at all the other positions in the sequence, enhancing the feature representation by learning the dependencies between the different positions. Each head processes the input independently in *MSA*, focusing on different parts of the sequence. While the *FFN* performs further nonlinear transformations on the output of the *MSA*.

For the encoding process of each Transformer encoder layer L, it can be expressed as:

(9) $$X_{p}^{\prime l}=MSA(LN(X_{p}^{l-1}))+X_{p}^{l-1},l=1,2,\ldots ,L$$

(10) $$X_{p}^{l}=FFN(LN(X_{p}^{\prime l}))+X_{p}^{\prime l}$$

where *LN* denotes the layer normalization operation, which is used to normalize the distribution of input features before self-attention and *FFN* at each layer, stabilizing the training process and improving the generalization ability of the model. $X_{p}^{\prime l}$ and $X_{p}^{l}$ represent the output features after *MSA* and *FFN*, respectively.

Moreover, in the process of physiological feature extraction by the encoder, to solve the problem of gradient vanishing in the deep network training, residual connections are added to ensure that the gradient can be better propagated to the shallower layers during backpropagation, thus helping the network to learn the feature representation more effectively.

For non-physiological data $X_{np}$, the process is the same as the physiological branch feature extraction process, whereas the inputs to the non-physiological branch are non-physiological data containing 11 dimensions of eye tracking (gaze point X and Y coordinates, pupil diameters of the left and right eyes), vehicle status (speed, acceleration, braking, lane offset, lane position, distance), and facial mood changes. These data are analyzed by Transformer’s *MSA* and *FFN*, and the learning process is enhanced by residual linkage to capture the long-term feature changes in the non-physiological data. After the above process, the final output features of the non-physiological branch are $X_{np}^{l}$.

After processing in this module, physiological and non-physiological features can be extracted, providing key feature representations for subsequent feature fusion and prediction tasks.

#### 2.5.3. AFF Module Based on Dynamic Weight Assignment

The input data $X_{p}$ and $X_{np}$ after going through the Dual-branch Transformer encoder module, the obtained $X_{p}^{l}$ and $X_{np}^{l}$ features containing complex timing dependencies and rich feature information. To effectively integrate these features for driving-related cognitive abilities prediction, an efficient fusion strategy needs to be designed to effectively integrate the key features contained in $X_{p}^{l}$ and $X_{np}^{l}$.

**Figure 3:**
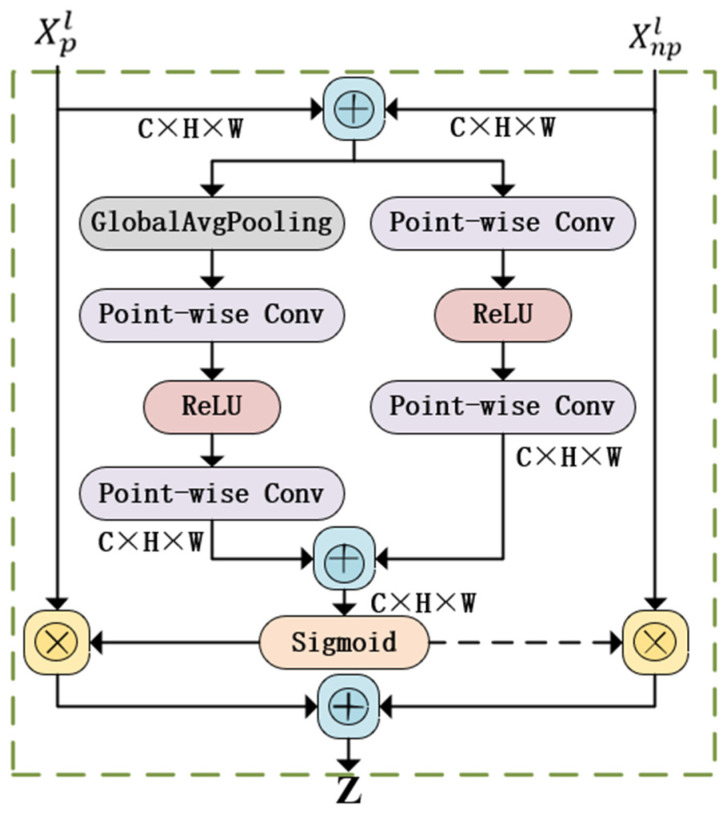
AFF network model.

Traditional feature fusion methods, such as simple splicing or weighted averaging, which are simple to design, usually ignore the interrelationships between different features and their relative contribution to the final prediction task. This approach cannot fully utilize all the available information features, especially in scenarios with complex interactions between features, which can easily lead to the omission of critical information or overemphasis on redundant features, which in turn affects the ACC of prediction. Therefore, this study introduces the AFF feature fusion module based on dynamic weight assignment, which optimizes the fusion process of different modal features and enhances the prediction capability of the model. In contrast, AFF adopts an approach based on the attention mechanism to dynamically adjust the contribution of different feature sources, to capture the complementary information between modalities more effectively and retain the key features. The AFF network model is shown in Figure 3.

In Figure 3, where $X_{p}^{l}$ and $X_{np}^{l}$ denotes the physiological and non-physiological features of the input, these two features go through the global extraction channel and the local extraction channel to obtain the attention weights, and then the obtained attention weights are multiplied with the input features, and finally aggregated to obtain the output Z, ⊗ denotes multiplication and ⊕ denotes addition.

In the AFF module based on dynamic weight assignment, the physiological features $X_{p}^{l}$ and non-physiological features $X_{np}^{l}$ output from the last layer of the Dual-branch transformer encoder is first split into two streams, the first stream initially integrates the physiological and non-physiological features through the summation operation and passes the summed features through the point-wise convolution layer. In this layer, the feature channels are optimized by linear transformations to adjust the relative importance between features and enhance their nonlinear representations to extract key global information features. The second stream multiplies $X_{p}^{l}$ and $X_{np}^{l}$ to extract key local information features. It uses a channel attention mechanism to dynamically weigh these features, adjusting their importance based on their relevance to the prediction task. Finally, the global and local features extracted by the two streams of addition and multiplication are fused to generate a comprehensive feature representation. The AFF process can be represented by Equation (10).

(11) $$F_{fusion}=\mathrm{Attention}(X_{p}^{l},X_{np}^{l})\cdot (X_{p}^{l}⊕X_{np}^{l})$$

where “Attention” denotes the weight assignment function computed by the attention mechanism, ⊕ denotes the fusion operation of the features, and $F_{fusion}$ is the final fused feature representation.

After processing in the above modules, a feature vector incorporating key information $F_{fusion}$ is finally generated, which is used for driving-related cognitive abilities prediction after the FC layer.

## 3. Experimental Results and Analysis

In this section, the effectiveness of the proposed driving-related cognitive abilities prediction model is evaluated through a series of experiments. Additionally, the methodology of this study is compared with existing machine learning algorithms and deep learning algorithms. Ablation experiments were also conducted to validate the effectiveness of the proposed method in this paper. The computer equipment used for the experiments in this study has the following configurations: an Intel(R) Core (TM) i7-8750H @ 2.20GHz, an NVIDIA GeForce GTX 3090 graphics card, Python 3.6.0 (64-bit), and PyTorch 1.5.0. The version of Tensorflow used for the environment is 2.6.0.

Under the computation power of a GTX 3090 GPU, with a batch size of 128, embedding dimension of 128, 12 layers, and 8 attention heads, the dual-branch Transformer feature fusion network used in this study (Baseline + Dual-Branch + AFF) has patch sizes of 4 × 4 and 11 × 4, with a parameter count of 7.90 MB, a computational cost of 25.72 GFLOPS, and an inference speed of 312 FPS. The baseline ViT model, with a patch size of 15 × 15, has a parameter count of 3.97 MB, a computational cost of 5.23 GFLOPS, and an inference speed of 344 FPS.

### 3.1. Data Set Segmentation

This study conducted a series of validation experiments using large-scale multimodal driving datasets. This dataset is described in detail in Section 2.1. In this section, the construction of the training–testing dataset will be described.

To further validate the stability and generalization ability of the model, this study employed subject-based cross-validation. In this approach, each subject was treated as an independent test set, with the remaining subjects’ data used for training. This process was repeated, and model evaluations were obtained for each sample. The overall performance of the model was then assessed by averaging the evaluation results across all samples. The specific implementation of this method follows the description in [46].

During the experiments, the parameters were set to 0.9 and 0.999 using the Adam optimizer, and the learning rate was corrected using StepLR, with the step size set to 1, gamma set to 0.5, initial learning rate set to 6e-4, and the batch size to 128. Based on our experience with different window lengths, we considered the length of 60 s, and a continuous window with a step size of 5 s was chosen for sample acquisition eventually generating a total of 31,187 samples, of which the sample sizes for each category were 6649, 7747, 12,134, and 4657, respectively.

### 3.2. Evaluation Metrics

The performance of the proposed network is evaluated using *ACC*, Confusion Matrix, and F1-score as evaluation metrics.

The *ACC* is calculated as follows: where False Positives (*FP*) and False Negatives (*FN*) are the negative and positive samples that are incorrectly predicted, while True Positives (*TP*) and True Negatives (*TN*) are the positive and negative samples that are correctly predicted, respectively.

(12) $$ACC=\frac{TP+TN}{TP+TN+FP+FN}$$

The confusion matrix, also known as the error matrix, is a standard format for representing *ACC* evaluations in the form of an n-row, n-column matrix. Each column of the confusion matrix represents the predicted category, and the total number of each column indicates the number of data predicted to be in that category; each row represents the true category to which the data belongs, and the total number of data in each row indicates the number of data instances in that category. The value in each column represents the number of real data predicted to be in that category. As can be seen, the confusion matrix portrays the combined effect of the model and gives a clear picture of the number of correct or incorrect predictions for each category.

The *F1-score* is calculated by balancing precision (*P*) and recall (*R*) using the following formula.

(13) $$F1=2\times \frac{P\times R}{P+R}$$

Through the evaluation of the model using the above metrics, the *F1-score*, which takes into account both precision and recall, effectively assesses the model’s performance in handling class imbalance issues. Compared to *ACC* alone, it provides more detailed performance information. The confusion matrix presents a detailed classification of the model’s predictions, revealing the model’s performance and biases across different categories, thus helping us identify and improve deficiencies in certain categories.

### 3.3. Comparison Experiment

The comparison models chosen for this study are Support Vector Machine (SVM) [47], Random Forest (RF) [48], LSTM [49], VGG-16 [50], ResNet [51], CNN-LSTM [35]. These models achieve excellent prediction results in the literature related to driving behavior assessment mentioned above. Therefore, these six algorithms are chosen in this study as the comparison algorithms for this experiment.

In this experiment, all comparison algorithms used the same dataset processing methods as in this paper, i.e., the same missing data padding, data normalization, etc.

**Figure 4:**
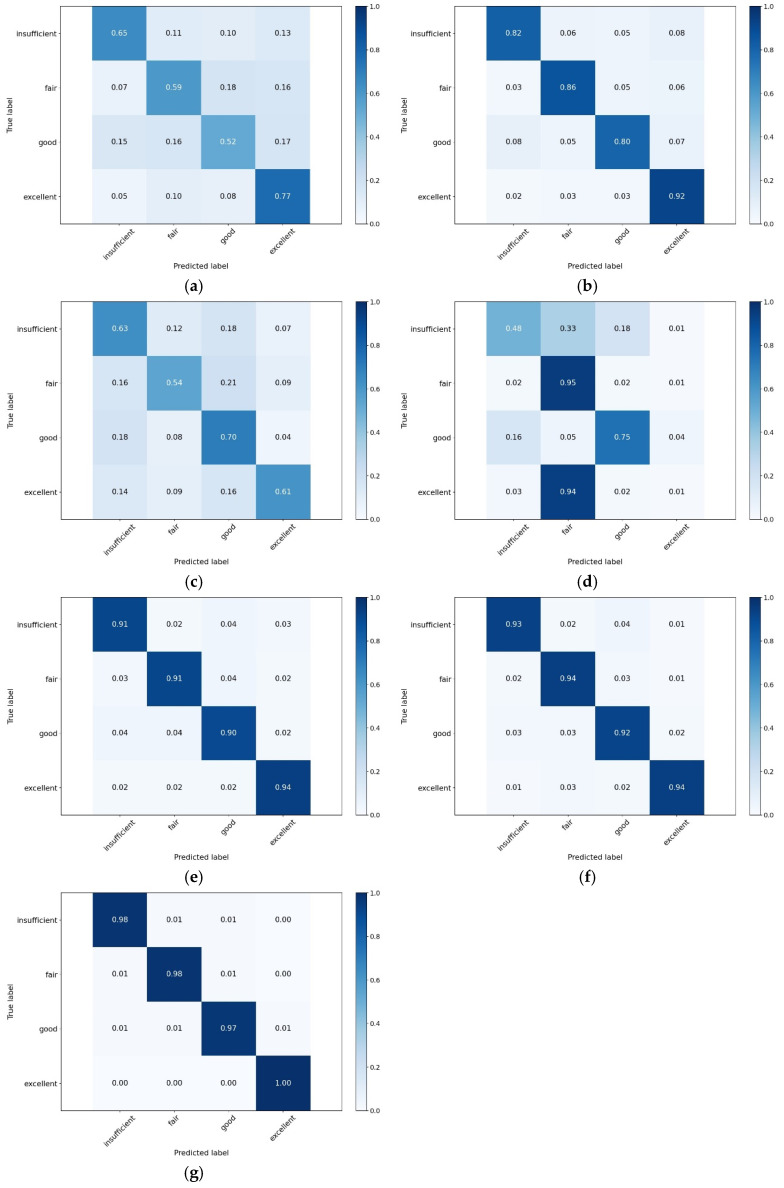
Confusion matrix results for SVM (**a**), RF (**b**), LSTM (**c**), VGG-16 (**d**), ResNet (**e**), CNN-LSTM (**f**), and our (**g**).

Table 4 shows the experimental results of all the algorithms using the ACC and F1-score metrics, and Figure 4 shows the experimental results of all the algorithms using the confusion matrix.

From the results of the comparison tests in the table, it can be observed that the model proposed in this study achieves high performance in terms of accuracy (Accuracy) and F1-score. Specifically, the model proposed in this study achieved an ACC rate of 0.9908 and an F1-score of 0.9832. Compared to SVM, RF, LSTM, VGG-16, ResNet, and CNN-LSTM, our model showed improvements in ACC of 36.13%, 13.25%, 33.47%, 32.09%, 4.37%, 3.11%, respectively, and improvements in F1-score of 38.31%, 13.28%, 34.64%, 49.00%, 5.68%, 5.30%, respectively. This demonstrates the superiority of the proposed model in predicting driving-related cognitive abilities. Both the CNN-LSTM and ResNet models achieved ACC rates exceeding 0.90. These models are frequently used for processing time-series data and effectively capture long-term and short-term features in the data. Specifically, CNN excels at extracting local features, while LSTM focuses on the temporal dependencies of sequential data, making CNN-LSTM highly effective in capturing complex temporal features. ResNet, based on deep residual networks, effectively addresses the vanishing gradient problem in deep networks through skip connections, thus demonstrating high predictive performance when processing multimodal data of driving behavior. In contrast, the RF model achieved an ACC and F1 score of 0.8583 and 0.8504, respectively. Although RF performs well with time-series data, it is less effective than our proposed model in predicting the cognitive abilities of drivers in tasks involving complex temporal features. The SVM, LSTM, and VGG-16 models demonstrated relatively lower performance, with ACC rates of 0.6295, 0.6561, and 0.6699, respectively. While SVM excels in handling high-dimensional data, it is limited in fusing multimodal and nonlinear data features. Although LSTM can handle temporal dependency data, it struggles to comprehensively capture spatial features in multimodal data when used alone. VGG-16, being primarily an image recognition model, lacks adaptability in handling multimodal time-series features, resulting in lower predictive ACC. In comparison, the dual-branch transformer network proposed in this study effectively divides multimodal driving data into physiological and non-physiological data and uses a dynamic weight-based feature fusion module to extract common features, thereby achieving effective prediction of driving-related cognitive abilities.

Figure 4 shows the confusion matrix experimental results of each algorithm in the driving-related cognitive abilities prediction task. From the results of the confusion matrix, it can be seen that the Dual-branch multimodal feature fusion network model proposed in this study has the best prediction results, with prediction ACC of 0.98, 0.98, 0.97, and 1.00 for each of the four categories, and more than 0.97 for each of the categories. Categorical performance on a few categories in particular (inadequate driving-related cognitive abilities and excellent driving-related cognitive abilities) also maintained a high level of stability. In contrast, the VGG-16 performed the worst in terms of driving-related cognitive abilities prediction. In particular, the prediction ACC for the categories of poor and excellent driving-related cognitive abilities were only 0.48 and 0.01, respectively. The VGG-16 predicted almost all the samples with excellent driving-related cognitive abilities into the category of average driving-related cognitive abilities, while the prediction ACC for the samples with poor driving-related cognitive abilities was only 0.48. This is mainly due to the imbalance of the distribution of driving-related cognitive abilities in reality, where the majority of the drivers have a moderate level of cognitive ability, with relatively few drivers at the extremes (highest and lowest). However, these minority groups, especially drivers with poor driving-related cognitive abilities, are at higher risk and require special attention. Therefore, this requires the model to have a high predictive power even when the sample is unbalanced. Results indicate that the proposed model, by effectively leveraging the Transformer architecture, can better capture and analyze the nuanced features of such data, thereby significantly improving prediction ACC.

### 3.4. Ablation Experiments

To verify the effect of the algorithm proposed in this study on the prediction ACC of driving-related cognitive abilities, ablation experiments will be conducted in this study and the results of the ablation experiments are shown in Table 5.

Table 5 shows the results of the ablation experiments performed in this study based on the Baseline. From the results of the ablation experiments, it can be seen that compared to the Baseline, the Dual-branch multimodal feature fusion network model (Baseline + Dual-Branch + AFF) proposed in this study achieved 0.9908 and 0.9832 in the metrics ACC and F1-Score, respectively, marking an improvement of 5.98% and 6.07% over the Baseline model, and an increase of 2.99% and 3.12% relative to using only the Baseline + Dual-Branch structure. This indicates that the dual-branch multimodal feature fusion model proposed in this study effectively enhanced the model’s ability to extract features from different modalities. Additionally, introducing the AFF module further optimized the integration process of features from different modalities, thereby improving the model’s prediction ACC. This improvement is attributed to the proposed dual-branch multimodal feature fusion network’s ability to adapt to the temporal distribution characteristics of different modal input data, reduce interference between different data sources, and fuse the output features of the dual-branch encoders at both local and global levels, effectively extracting the nonlinear time-series features of physiological and non-physiological data, and enhancing the model’s predictive precision.

**Figure 5:**
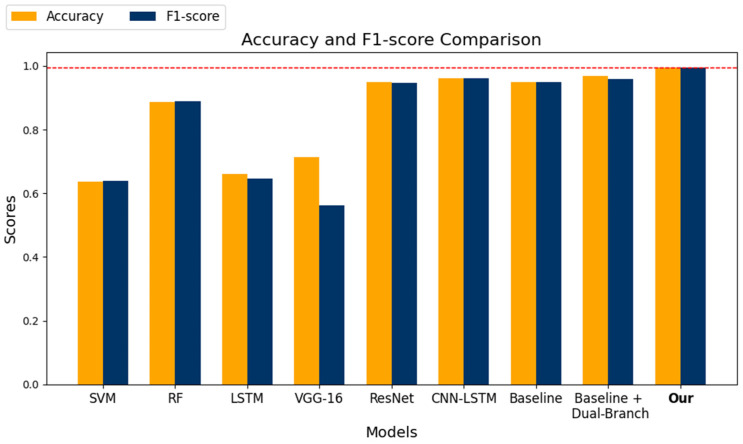
Comparison of experimental visualization results.

Figure 5 shows a comparison of the experimental results in terms of Accuracy and F1-score, highlighting the improvements of our model over the baseline and comparison models. The results demonstrate that our model outperforms the baseline model as well as other comparison models, exhibiting superior performance.

## 4. Conclusions

Driving relies on multiple cognitive abilities. To address the relationships between these abilities and driving performance, as well as to reduce potential information loss or redundancy in multimodal data processing, this study proposes a high-precision framework for predicting driver cognitive abilities. The framework consists of four modules: data preprocessing, cognitive ability extraction, label generation, and a cognitive ability network model. The extraction and label generation modules analyze driving videos to identify ten cognitive components critical for decision-making, vehicle control, and psychological regulation. These components are quantified and weighted to generate cognitive ability labels.

In the cognitive ability network model, this study innovatively applies a Transformer structure to extract both physiological and non-physiological multimodal features, enhancing the model’s capacity for feature extraction and improving prediction accuracy (ACC). The experimental results demonstrate that the proposed framework achieves ACC and F1-scores of 0.9908 and 0.9832, respectively, outperforming RF, SVM, LSTM, ResNet, CNN-LSTM, and VGG-16 models. This prediction model could be applied in driving assessments for licensing, training programs to address individual weaknesses, and personalized driving assistance systems to offer real-time support and warnings. It provides a novel approach to driving risk assessment.

While this study successfully constructs a multimodal-based framework for predicting driving-related cognitive abilities, several limitations remain. The use of video-based analysis for driver behavior introduces the potential for human error, which could affect the objectivity and accuracy of the data. Future work could focus on automating key indicator extraction to enhance reliability. Additionally, factors such as driver experience (e.g., years licensed) may impact ability assessments and should be considered in future studies. The study’s limited focus on younger and older drivers restricts its applicability across all age groups, with insufficient data on middle-aged drivers (aged 27 to 60). Furthermore, the complexity of multimodal data collection may make device usage cumbersome, affecting the framework’s practical feasibility. Future research could focus on optimizing data acquisition devices by selecting a small number of high-precision sensors to reduce unnecessary hardware requirements, thus simplifying the data collection process and enhancing the system’s operability and practicality. Additionally, the introduction of lightweight deep learning models could reduce the number of model parameters and computational complexity, decreasing reliance on computational resources, and improving the system’s real-time responsiveness, computational efficiency, and deployability.

## Figures and Tables

**Table 1:** Relevant studies on driving behavior assessment.

Reference	Subject of Study	Data	Assessment Method	Performance (ACC)
Stanisław, et al. [20]	Reaction Speed	Accelerator Pedal, Brakes, Steering Wheel	Statistical Analysis	-
Broadbent, et al. [24]	Working Memory Capacity	FNIRS, Eye Tracker	Statistical Analysis	-
Moran, et al. [9]	Hazard Perception Ability	Cognitive Tests, Hazard Perception Test (HPT)	Statistical Analysis	-
Chen, et al. [17]	Distraction	Eye, Vehicle, Physiological	Deep-CNN	99.78%
Mohammed, et al. [18]	Distraction	Driving Image Data	Semi-supervised Lightweight Hybrid VIT	95.43%
Mou, et al. [32]	Distraction	Eye, Vehicle, Physiological	CNN-Trans-SA	99.80%
Arvin, et al. [33]	Distraction	Vehicle Motion, Cabin Video, Environment	1DCNN-LSTM	95.45%
Mou, et al. [34]	Stress	Eye Data, Vehicle Data, Environmental Data	CNN-LSTM	95.5%
Arefnezhad, et al. [35]	Drowsiness	Vehicle	CNN-LSTM	96.0%
Vyas, et al. [36]	Driving Safety	Accelerometer, Gyroscope, and GPS	Trans-DBC	95.38%

**Table 2:** Driving-related cognitive abilities extraction method.

Dimension	Driving-Related Cognitive Abilities	Task Scenario	Data Sources and Extraction Method
Cognitive decision-making	Attention Allocation Ability	Completing secondary tasks Completing secondary tasks Passing through an intersection	Record the number of times secondary tasks were avoided
	Working Memory Capacity		ACC of completing secondary tasks
	Anticipatory Ability		Accurate anticipation of traffic signals
Vehicle Control	Reaction Ability	Vehicle malfunction acceleration, a car suddenly entering the road from the left	Time from recognizing an emergency to execution (e.g., braking or evading)
	Judgment Ability		Record evasion strategies and extent of vehicle collision
	Processing Speed	Completing secondary tasks	Reaction time to complete secondary tasks
	Perception Ability	Passing through an intersection	Whether the line is crossed during red light waiting
Psychological regulation	Stress Resistance	Before and after simulated driving tasks	NASA scale score
	Anxiety Level		Anxiety scale score
	Type A/B Personality		Type A/B personality scale score

**Table 4:** ACC and F1-score experimental results for all compared algorithms.

Comparison Algorithm	Epoch	ACC	F1-Score
SVM	-	0.6295	0.6001
RF	-	0.8583	0.8504
LSTM	100	0.6561	0.6368
VGG-16	100	0.6699	0.4932
ResNet	100	0.9471	0.9264
CNN-LSTM	100	0.9597	0.9302
Our	100	0.9908	0.9832

**Table 5:** Ablation experiments.

Experiments	Epoch	ACC	F1-Score
Baseline	100	0.9310	0.9225
Baseline + Dual-Branch	100	0.9609	0.9520
Baseline + Dual-Branch + AFF (our)	100	0.9908	0.9832

## Data Availability

Data are contained within the article.
